# Psychological Features of Takotsubo Cardiomyopathy: Report of Four Cases

**Published:** 2017-04

**Authors:** Yaser Jenab, Seyedeh Roghaieh Hashemi, Neda Ghaffari-Marandi, Hoda Zafarghandi, Nazila Shahmansouri

**Affiliations:** 1 *Tehran Heart Center, Tehran University of Medical Sciences, Tehran, Iran.*; 2 *Shahid Beheshti University, Tehran, Iran.*; 3 *Tehran University, Tehran, Iran.*

**Keywords:** *Takotsubo cardiomyopathy*, *Psychiatry*, *Women*, *Stress, psychological*, *MMPI*

## Abstract

Takotsubo or stress-induced cardiomyopathy is a cardiomyopathy in which the patient has a sudden onset, reversible left ventricular systolic dysfunction without any significant coronary artery disease. Four women, who were at a mean age of 64 years and suffered from chest pain exacerbated by emotional stress, were admitted as cases of acute coronary syndrome and were completely evaluated through precise history taking, physical examination, and ECG. Coronary angiography or coronary multidetector computed tomography was used to exclude significant coronary artery disease. In these patients with confirmed Takotsubo cardiomyopathy, in addition to the Diagnostic and Statistical Manual of the American Psychiatric Association (DSM-IV) criteria, a 71-item form of the Minnesota Multiphasic Personality Inventory (MMPI)-Mini-Mult-was employed for psychological assessment. The main common elevated scale was hypochondriasis. Individuals with high scores on this scale are obsessed with themselves, especially in regard to their body, and often use their disease symptoms in order to manipulate others. They are mainly passive aggressive, critical, and demanding, which stems from their lack of effective verbal abilities as a means of communication, specifically when it comes to anger or hostility expression. To the best of our knowledge, there is no available study evaluating patients with Takotsubo cardiomyopathy using the Mini-Mult questionnaire for psychological assessment.

## Introduction

Takotsubo or stress-induced cardiomyopathy (TCM), also known as broken heart syndrome, is a cardiomyopathy in which the patient has a sudden onset, reversible left ventricular systolic dysfunction.^1^ This disorder develops without any significant coronary artery disease (CAD). Sudden onset of intense emotional or physical stresses can spark off this syndrome. This condition is more common amongst post-menopausal women. The prevalence of TCM in previous studies was between 0.7% and 2.5% of cases presenting with suspected acute coronary syndrome. Chief complaints on presentation include chest pain and dyspnea, which renders the diagnosis more complex.^1^

In previous studies, the prevalence of anxiety or depression was reported at 56% and 48% in these patients. The most probable predisposing factor is believed to be chronic anxiety disorders; therefore, it has been recently suggested that psychological factors might contribute to the pathophysiology of TCM.^2^ Mood disorders are even more common in these groups of patients than are other cardiac risk factors such as diabetes and smoking.^1^ We herein present the personality features of 4 patients with TCM. 

## Case Report

Four patients with a confirmed diagnosis of TCM admitted to our center between September 2010 and August 2010 were included. The patients were admitted as cases of acute coronary syndrome and were treated accordingly. They were evaluated by precise history taking, physical examination, and electrocardiography. Coronary angiography or coronary multi- detector computed tomography was used to exclude significant CAD. The diagnosis of TCM was made in accordance with the Mayo Clinic’s criteria.^3^ In addition to the Diagnostic and Statistical Manual of the American Psychiatric Association (DSM-IV) criteria,^4^ a 71-item form of Minnesota Multiphasic Personality Inventory (MMPI) - Mini-Mult was employed for psychological assessment.^4^ The Mini-Mult has been shown to be reliable and highly related to the standard MMPI.^5^ This test has been validated in different previous studies and has also been validated in Iran.^6^ Raw scores were converted to T scores via the Kincannon conversion table.

**Figure 1:**
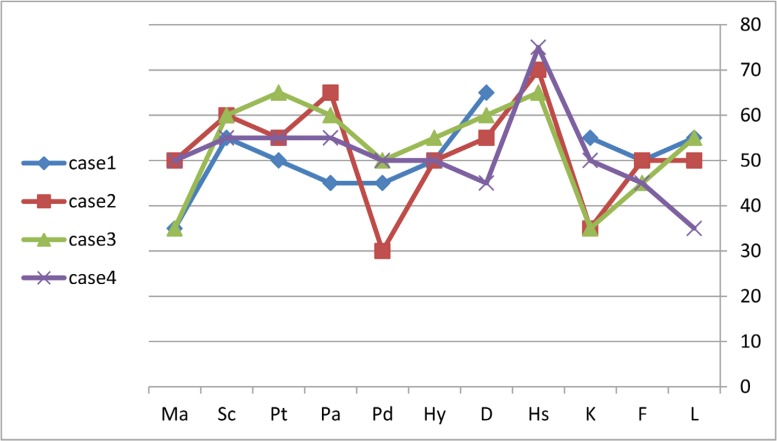
Mini-Mult scores of the patients with Takotsubo cardiomyopathy.

Before the interview, the patients were fully informed about the aim of the research. Four patients who met the criteria of TCM were assessed. The basic characteristics of these patients are presented in Table 1. According to the Mini-Mult scores (Figure 1), case 1 had no personality disorder. The patient did not verbalize her problems fully and clearly. It seemed that the patient had situational depressive reactions, somatic complaints under stressful situations, hidden anxiety, and even psychotic attitudes. Based on the DSM-IV interview, she was diagnosed as having a major depressive disorder.

Case 2 had situational depressive reactions, anxiety, paranoia, and indirect expression of aggression. Her performance was below the standard. She had psychotic attitudes and somatic complaints under stressful conditions (Figure 1). According to the DSM-IV, she was diagnosed with a major depression disorder.

Case 3 showed situational depressive reactions, anxiety, health concerns and psychotic attitudes under some conditions, sensitivity, irritability, and somatic complaints under stressful situations. She also had a generalized anxiety disorder.

Finally, case 4 had no personality disorder. She had situational depressive reactions and somatic complaints under stressful conditions. Pessimistic and psychotic attitudes under difficult conditions were also reported. Additionally, she tended to hide her anxiety, and her performance level was low. According to the DSM-IV, she had no major psychiatric disorder.

L, Lie; F, Infrequency; K, Correction; Hs, Hypochondriasis; D, Depression; HY, Hysteria; Pd, Psychopathic deviation; Pa, Paranoia; Pt, Psychasthenia; Sc, Schizophrenia; Ma, Hypomania

**Table 1:** Characteristics of the study patients

	Case 1	Case 2	Case 3	Case 4
Age (y)	58	58	51	60
Sex	Female	Female	Female	Female
PMH	DM, HLP, HTN	TCM		
Clinical symptom	chest pain, dyspnea	chest pain, dyspnea, agitation	chest pain	chest pain, dyspnea, vomiting
Exacerbating factor	argument with her son-in-law	extreme happiness, excitement, dancing	witnessed the sudden death of her nephew	sudden death of her niece
ECG findings	T inversion in anteroseptal leads	T inversion in anterior, inferior and lateral leads, QT prolongation (535 msec)	normal	T inversion in anteroseptal, anterior and inferior leads
Peak hsTnT (ng/L)	129.7	213.0	103.3	242.0
CAG/MDCT findings	normal coronary Arteries	normal coronary Arteries	minimal CAD	minimal CAD
Admission LVEF	30%	23%	45%	30%
Follow-up LVEF	55%	55%	55%	55%

PMH, Past medical history; DM, Diabetes mellitus; HLP, Hyperlipidemia; HTN, Hypertension; TCM, Takotsubo cardiomyopathy; hsTnT, High-sensitive troponin T (normal < 24 ng/L); LVEF, Left ventricular ejection fraction; MDCT, Multi-detector computed tomography; CAG, Coronary angiography; CAD, Coronary artery disease

## Discussion

TCM usually occurs in post-menopausal women. About 94% of TCM cases have been found in women at a mean age ranging from 52 to 80 years. In the majority of the patients, the clinical presentation is indistinguishable from that of acute coronary syndrome, including chest pain and dyspnea.^1^ Sympathetic over-activity is also another known feature in TCM which is similar to what we found in our patients. The tendency not to share negative emotions such as those aroused by a stressful situation is believed to be associated with exaggerated sympathetic and cardiovascular responses.^7^

Our patients were exposed to a stressful situation, which along with their inability to express emotions and their somatizing tendencies, may have rendered them susceptible to TCM. However, individuals are liable to somatize when faced with an inner or outer situation that overwhelms their habitual psychological ways of coping; the body reacts to a psychological threat as though it were a physiological one.^8^

Our findings also suggest that a psychosomatic component is present here. It seems that our patients’ body reacted to a psychological threat as though it were a physiological one. Half of psychiatric patients initially visited by medical doctors complain of somatic symptoms^9^ and most of them are depressed.^10^

Denial, suppression, and repression are the most frequent defense mechanisms that these patients drawn upon to control their emotions and thoughts.^11^ It has been postulated that this is due to patients’ unawareness of their emotional states in threatening situations.^8^ It has always been emphasized that psychosomatic patients fail to fully express their own feelings and their conditions will exacerbate when they cannot engage with their distress and feelings.^12^

If the loss is partial, the patient - though aware of some feeling - is unable to describe it well (Mudd JO, Kapur NK, Champion HC, Schulman SP, Wittstein IS. Patients with stress-induced (takotsubo) cardiomyopathy have an increased prevalence of mood disorders and antidepressant use compared to patients with acute myocardial infarction. J Card Fail 2007;13:S2-S176). These patients are unable to reflect either on their own mental world or that of the others. They regress to a preverbal and preconceptual state in which perceptions and sensations predominate.^13^

The main common elevated scale is hypochondriasis. Individuals with high scores on this scale are obsessed with themselves, especially with respect to their body, and often use their disease symptoms in order to manipulate others. They are mainly passive aggressive, critical, and demanding, which-in a sense-also refers to their lack of effective verbal abilities as a means of communication specifically when it comes to anger or hostility expression.^14^

Two of our patients had experienced a loss, which is consistent with another study suggesting that loss experiences through death or separation from someone close (e.g., son, father/mother, husband, and friend) were the most prevalent emotional triggers in both stress cardiomyopathy and acute myocardial infarction groups.^7^

Although there are a few small investigations in the existing literature reporting a link between psychological factors and TCM, the personality of these patients has yet to be fully elucidated.^1^ Based on these results, further cross-sectional studies with a larger sample size or longitude studies may assist in confirming and generalizing these findings. 

## Conclusion

Psychological disorders are the most probable predisposing factors in TCM. To the best of our knowledge, there is no available study evaluating patients with TCM using the Mini-Mult questionnaire for psychological assessment.

## Notes:

***This paper should be cited as: ***Jenab Y, Hashemi SR, Ghaffari-Marandi N, Zafarghandi H, Shahmansouri N. Psychological Features of Takotsubo Cardiomyopathy: Report of Four Cases. J Teh Univ Heart Ctr 2017;12(2):95-98.
